# 

**Published:** 1916-01-22

**Authors:** 


### PASSING EVENTS AND TOPICS.

### The M.A.B. and its Bacteriological Adviser.

PROFESSOR G. SIMS WOODHEAD, M.A., M.D., C.M., F.R.S.E., has been reappointed by the Metropolitan Asylums Board as their consultative bacteriological adviser at a salary of £105 per annum.

### North Ormesby Hospital.

WE are glad to hear that Mr. Arthur Williams, secretary of the North Ormesby Hospital, is able to resume his work at the hospital after a severe illness lasting upwards of three months.

### King Edward's Hospital Fund for London.

A MEETING of the General Council of King Edward's Hospital Fund for London was held on January 10, at the offices of the Fund, 7 Walbrook, E.C., for the purpose of formally adopting the resolutions providing for the work of the Fund for 1916, which were approved by the Governors and General Council at the meeting held at St. James's Palace on December 16, 1915.

### X-ray Cars for the Front.

ON January 27 a mass meeting will be held at the Queen's Hall, London, on behalf of Countess Helena Gleichen's scheme for radiograph cars at the Front. An assembly of representatives of the various branches of women war-workers will march in procession from the Embankment to the Queen's Hall.

### Surgical Rubber and the Blockade.

ACCORDING to the *Times* correspondent at Washington, efforts are being made in certain quarters to work up an agitation against our refusal to allow rubber gloves and rubber sheets to be sent by the American Red Cross to Germany. It is alleged by Mr. von Briesen, the chairman of the American Physicians' Expedition Committee, that the death of an American nurse in Germany was due to infection by uncovered hands, and hence, indirectly, to our blockade. Whatever merits this story may have, our action regarding surgical rubber is troubling the American Red Cross, and representations have been made in appropriate quarters.

### Manchester Royal Infirmary.

MAJOR R. A. NEEDHAM, M.B., Indian Medical Service, who is among the officers upon whom the D.S.O. has been conferred, is an ex-member of the surgical staff of the Manchester Royal Infirmary. Dr. Mabel May, who has been appointed to the Women's Maternity Unit for Russia, organised by the National Union of Women's Suffrage Societies, is also a member of the infirmary staff. Last March she received permission from the board of management to volunteer for work in Serbia, and for nine months she was with Mrs. St. Clair Stobart's unit at Nish. Dr. May, who has already taken up her new appointment, was prominently identified in Manchester with the establishment of the hospital for babies.

### Gifts and Bequests.

Mr. F. Doubleday, of Emneth, Norfolk, left £800 to the Wisbech Cottage Hospital.

Mr. Alfred Bernstein Amber left £20 each to King Edward's Hospital Fund and the London Hospital.

Mr. T. P. Bailey, of Wallgaston, Berkeley, Glos., left £400 to the Berkeley Hospital and £300 to the Bristol Eye Hospital.

The Royal Dental Hospital, Leicester Square, has received a donation of £2,000 from the trustees of the late Miss Harker-Smith.

Mrs. Grace Jamesina Harrison, of Durham, left £100 each to the Blind Asylum Infirmary, the Eye Infirmary, Durham, and the Monkwearmouth Hospital.

The Chelsea Hospital for Women has received a legacy of £1,000 from the late Mr. John Samuel White, also £250, part of the legacy bequeathed by the late Miss Jane Simmons.

Mr. Edward John Walford, of Langford Place, St. John's Wood, left £1,000 to the London Hospital for a bed in memory of his son, Lieutenant Leonard N. Walford, 12th London Regiment, who was killed in the recent terrible battle at Hill 60.

Mrs. Anna Maria Addis, of the Mill House, Pixham, Dorking, bequeathed a sum sufficient to endow a bed for an adult and a cot for a child in the Dorking Cottage Hospital; the bulk of her estate—of the value of over twelve thousand pounds—she left to Guy's Hospital for the out-patients' department.

### THE BOOK MARKET.

LIST OF NEW BOOKS RECEIVED FROM THE FOLLOWING PUBLISHERS:—

T. B. Browne, Ltd.: "The Advertiser's A B C," 1916. 10s. 6d.

J. and A. Churchill: "The Primary Lung Focus of Tuberculosis in Children." By Dr. Anthon Ghon, translated by D. Barty King, M.A., M.D. 10s. 6d. net.

Humphrey Milford, Oxford University Press: "The Pathology and Treatment of the So-called Nervous Asthma." By J. B. Berkart, M.D. 2s. 6d. net.

John Murray: "Rural Sanitation in the Tropics." By Malcolm Watson, M.D. 12s. net.

Oxford Medical Press: "Instinct and Intelligence." By N. C. Macnamara, F.R.C.S. 6s. net.

Geo. Philip and Son, Ltd.: "Philip's Popular Mannikin." By W. S. Furneaux. 3s. 6d. net.

G. P. Putnam's Sons: "Anatomy and Physiology for Nurses." By Amy E. Pope. Second Edition. 7s. 6d. net. "A Quiz Book of Nursing." By Amy E. Pope and Thirza A. Pope. Second Edition. 7s. 6d. net.

W. Rider and Son, Ltd.: "Health for Young and Old." By A. T. Schofield, M.D. 2s. net.

The St. Catherine Press: "The Wounded French Soldier." By Dion C. Calthrop. 1s. 6d. net.

The Scientific Press, Ltd., London: "The *Nursing Mirror* Pocket Encyclopædia and Diary," 1916. 6d. net. "The Midwife's Pocket Encyclopædia and Diary," 1916. 6d. net.

Jas. Willing, Ltd.: "Willing's Press Guide," 1916. 1s.

J. Wright and Sons, Ltd., Bristol: "First Aid to the Injured and Sick." By F. J. Warwick, B.A., M.B., etc., and A. C. Tunstall, M.D., C.M., etc. Ninth Edition. 1s. net. Warwick and Tunstall's "Questions on First Aid." 6d. net.

RATES OF SUBSCRIPTION : Twelve months, 6/6; Six months, 4/-; Three months, 2/-, post free to any address in the United Kingdom. Abroad, Twelve months, 8/8; Six months, 5/-; Three months, 2/6, post free.—Address The Subscription Dept., "THE HOSPITAL," The Hospital Building, 28/29 Southampton Street, Strand, London, W.C.

